# Supplementary material for: Identification of Antibody-Mediated Hydrolysis Sites of Oligopeptides Corresponding to the SARS-CoV-2 S-Protein by MALDI-TOF Mass Spectrometry
Source: Int J Mol Sci. 2023 Sep 20;24(18):14342. doi: 10.3390/ijms241814342 (PMC10531968; doi:10.3390/ijms241814342)
Supplement: Supplementary file 1 [file ijms-24-14342-s001.zip › ijms-2591205-supplementary.pdf]

International Journal of
Molecular Sciences

MDPI

Supplementary data

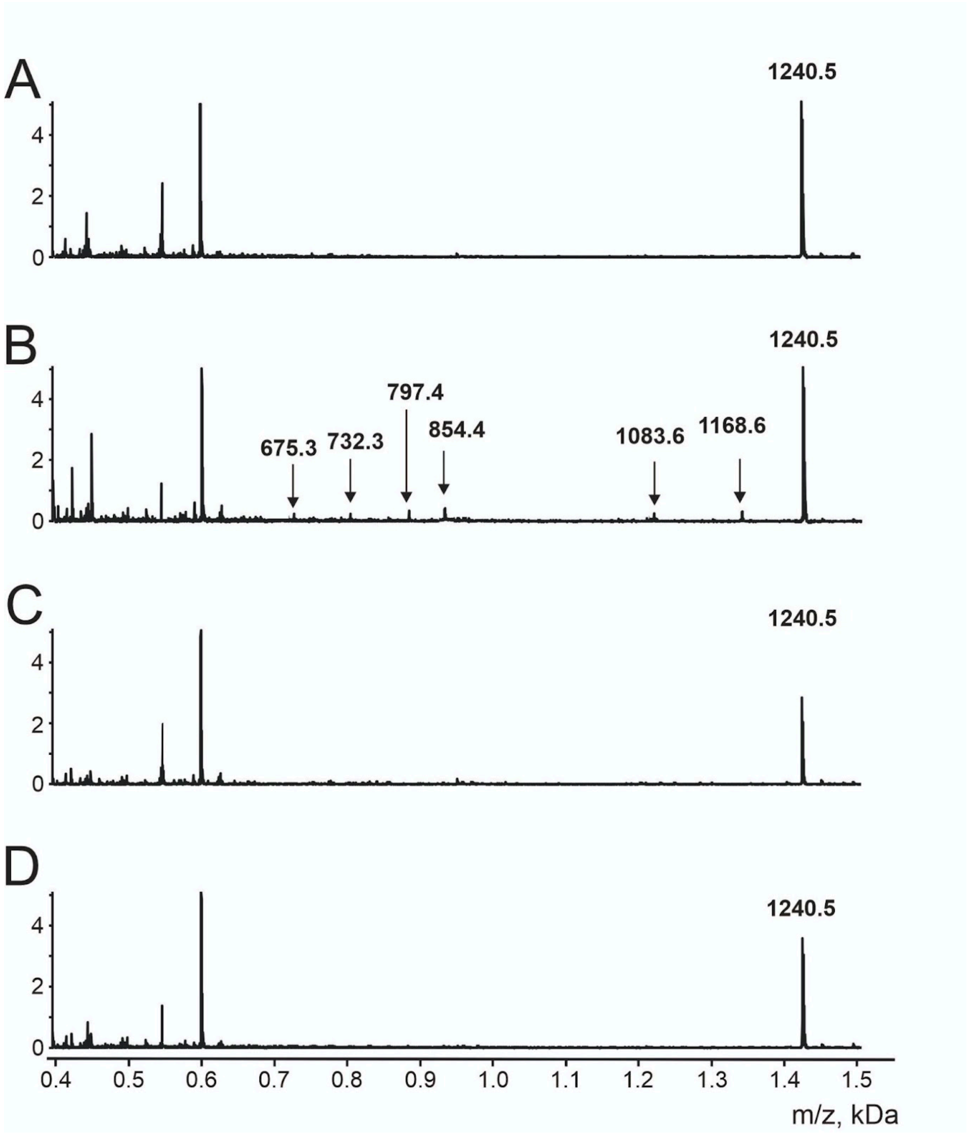

Figure S1. MALDI-TOF MS spectra of hydrolysis products of the AR oligopeptide by S-IgG antibodies of Con+Vac group (A), Con group (B), and Vac group (C) patients. MALDI-TOF MS of the intact oligopeptide (D). The 1240.5 peak corresponds to the intact AR oligopeptide. The peaks with m/z less than 0.6 in the spectrum of D correspond to the matrix components.

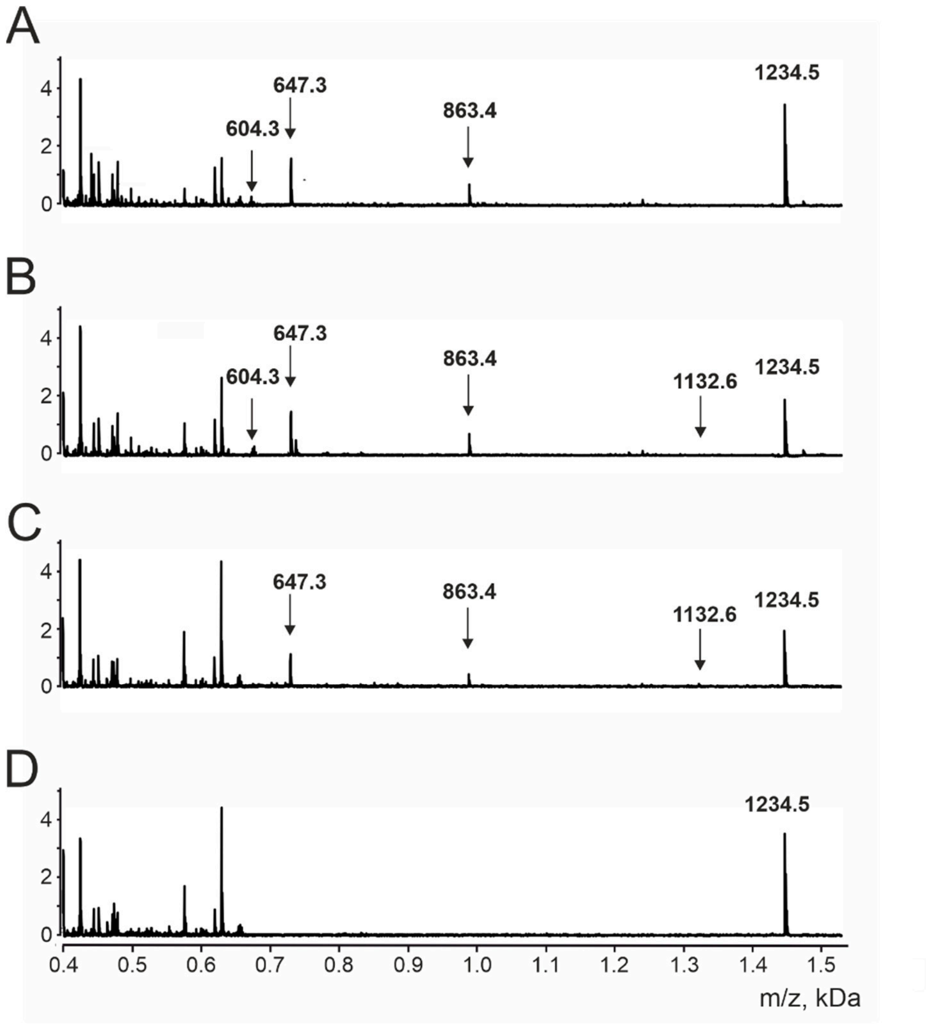

Figure S2. MALDI-TOF MS spectra of hydrolysis products of the TK oligopeptide by S-IgG antibodies of Con+Vac group (A), Con group (B), and Vac group (C) patients. MALDI-TOF MS of the intact oligopeptide (D). The 1234.5 peak corresponds to the intact TK oligopeptide: TGTGVVLTESNKK. The peaks with m/z less than 0.6 in the spectrum of D correspond to the matrix components.

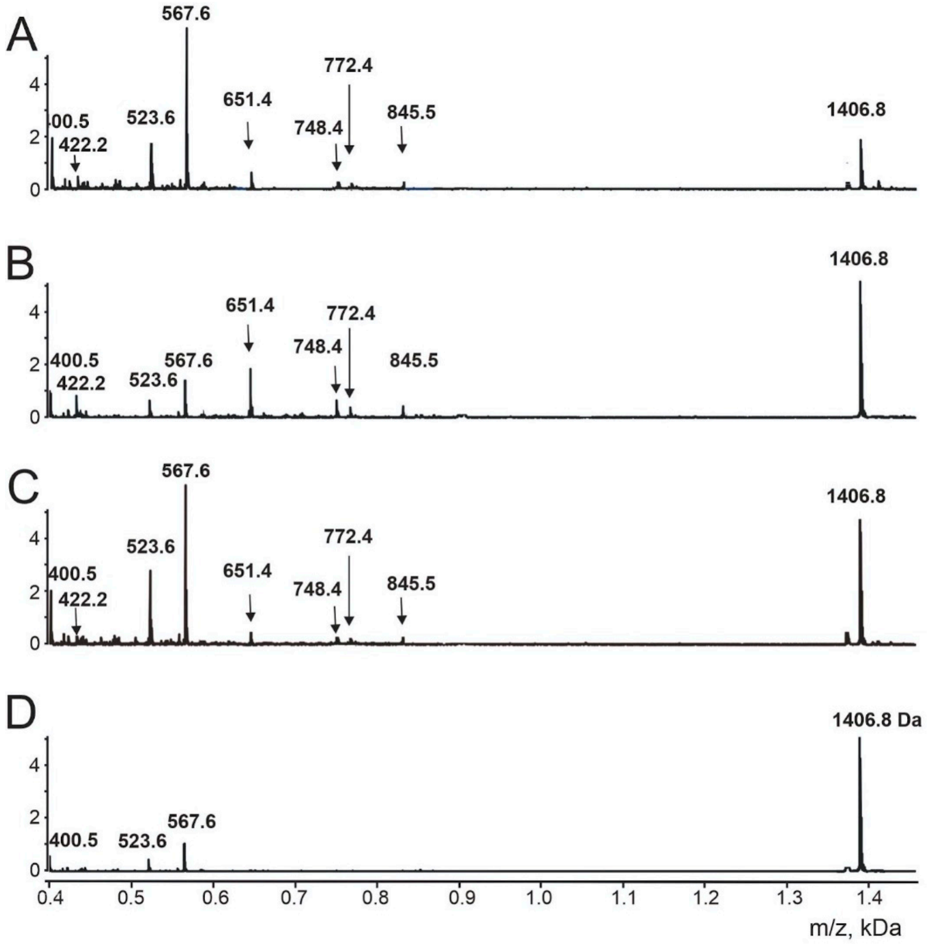

Figure S3. MALDI-TOF MS spectra of hydrolysis products of the QG oligopeptide by S-IgG antibodies from Con+Vac (A), Con (B), and Vac (C) patients. MALDI-TOF MS of the intact oligopeptide (D). The peaks with m/z less than 0.6 in the spectrum of D correspond to the matrix components.

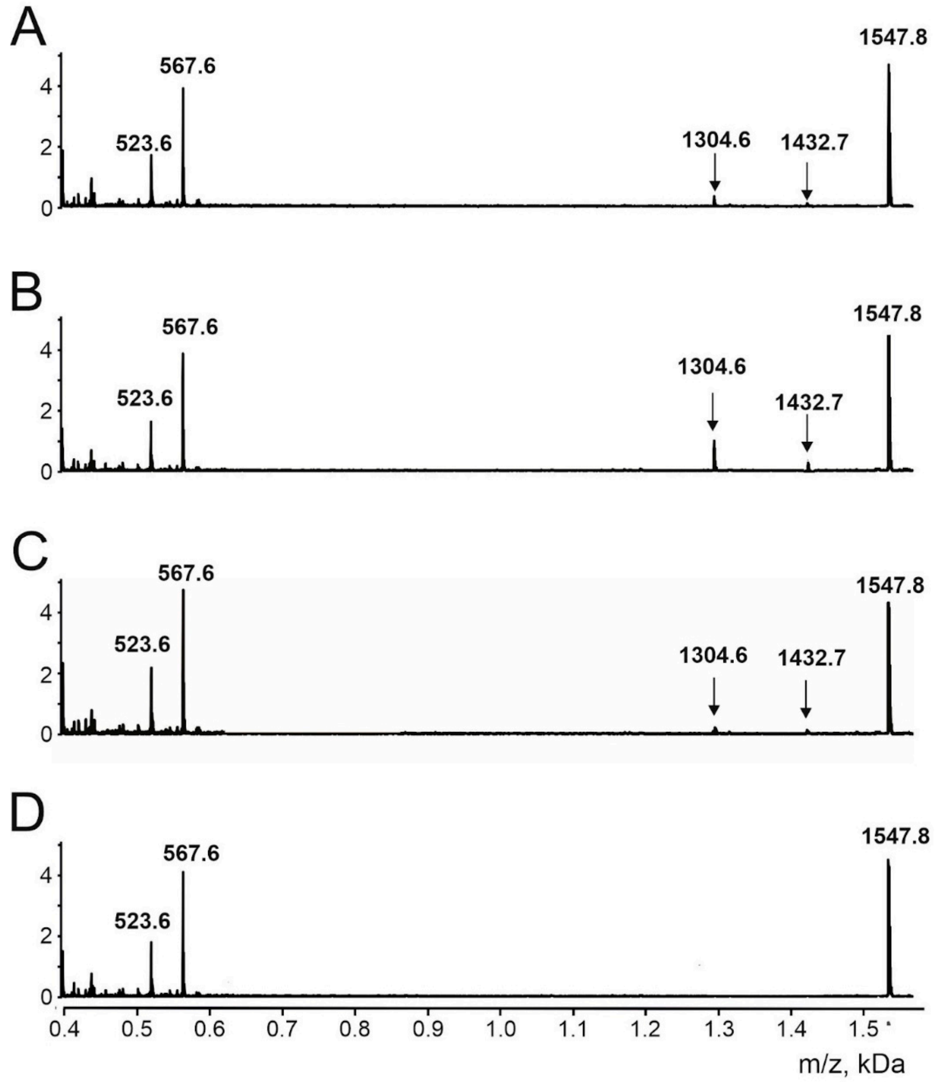

Figure S4. MALDI-TOF MS spectra of hydrolysis products of the SN oligopeptide by S-IgG antibodies of Con+Vac group (A), Con group (B), and Vac group (C) patients. MALDI-TOF MS of the intact oligopeptide (D). The peaks with m/z less than 0.6 in the spectrum of D correspond to the matrix components.

Table S1. A comparison of the MALDI-TOF spectrometry peaks obtained and molecular masses calculated for the possible fragments of the AR oligopeptide.

Oligopeptide fragment	Calculated value, kDa	Experimental value m/z, kDa	Hydrolysis site
TNGTKR	675.37	675.3	G6↓T7
GTNGTKR	732.39	732.3	S5↓G6
AIHVSGTN	797.40	797.4	N8↓G9
AIHVSGTNG	854.42	854.4	G9↓T10
AIHVSGTNGTK	1083.57	1083.6	K11↓R12
IHVSGTNGTKR	1168.63	1168.6	A1↓I2

Table S2. A comparison of the MALDI-TOF spectrometry peaks obtained and molecular masses calculated for the possible fragments of the TK oligopeptide.

Oligopeptide fragment	Calculated value, kDa	Experimental value m/z, kDa	Hydrolysis site
GVLTES	604.31	604.3	T3↓G4, S9↓N10
TGTGVLT	647.35	647.3	T7↓E8
TGTGVLTES	863.42	863.4	S9↓N10
GTGVLTESNKK	1132.61	1132.6	T1↓G2

Table S3. A comparison of the MALDI-TOF spectrometry peaks obtained and molecular masses calculated for the possible fragments of the QG oligopeptide.

Oligopeptide fragment	Formula	Calculated value, kDa	Experimental value m/z, kDa	Hydrolysis site
QIYKTPP	m/1	845.46	845.5	P7↓I8
PPIKDFG	m/1	772.41	772.4	T5↓P6
QIYKTP	m/1	748.41	748.4	P6↓P7
QIYKT	m/1	651.36	651.4	T5↓P6
QIYKTPP	m/2	422.73	422.2	P7↓I8

Table S4. A comparison of the MALDI-TOF spectrometry peaks obtained and molecular masses calculated for the possible fragments of the SN oligopeptide.

Oligopeptide fragment	Calculated value, kDa	Experimental value m/z, kDa	Hydrolysis site
SFKEELDKYF	1304.63	1304.6	F10↓K11
SFKEELDKYFK	1432.72	1432.7	K11↓N12
